# Supplementary figures and images for: Effects of antibiotic treatment on microbiota, viral transmission and viral pathogenesis of MoMuLV ts1 infected BALB/c mice
Source: PLoS One. 2022 Jan 21;17(1):e0261689. doi: 10.1371/journal.pone.0261689 (PMC8782509; doi:10.1371/journal.pone.0261689)

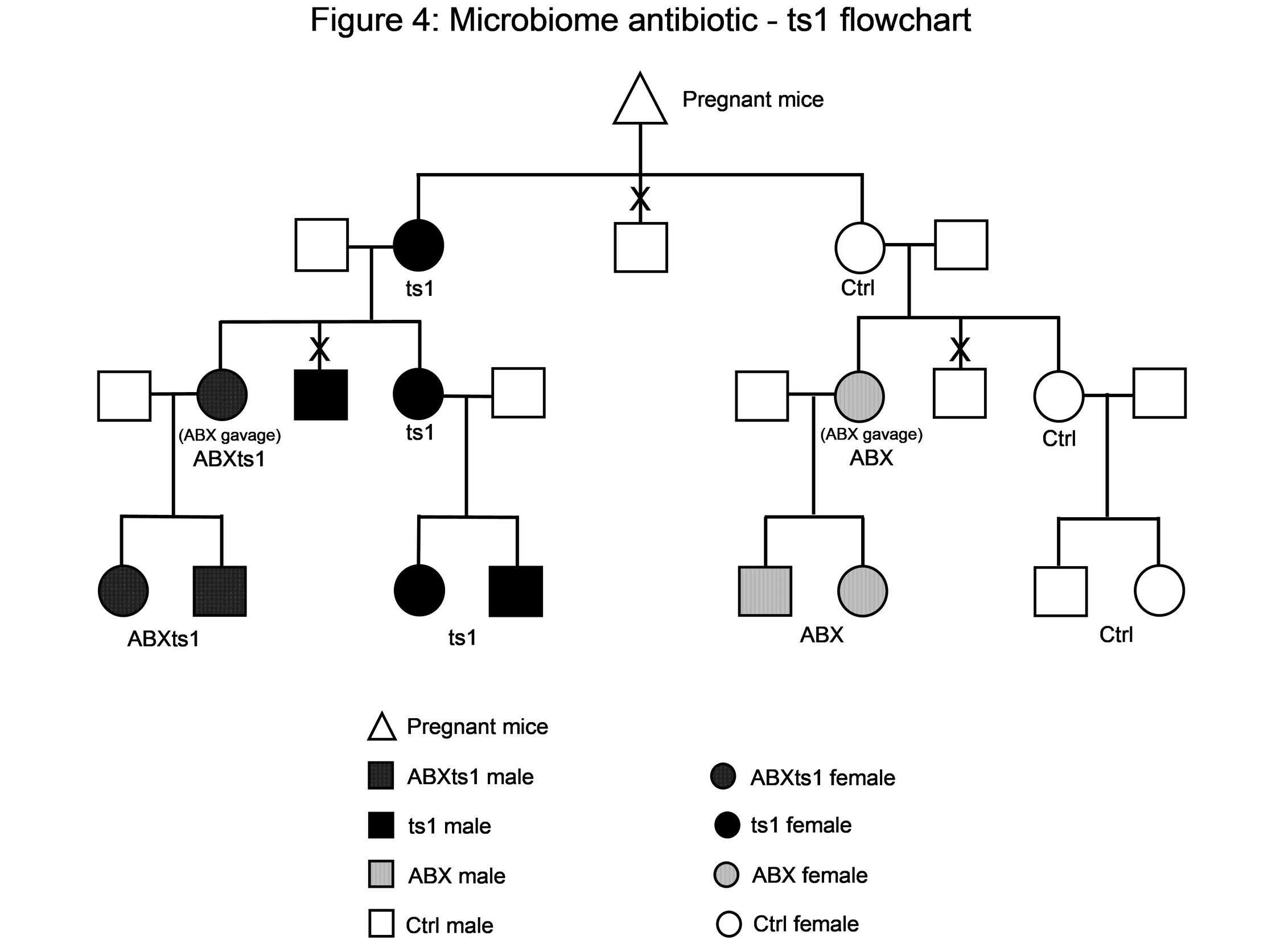

Supplement: S1 Fig — Experimental design illustrating the inoculation, transmission of ts1 virus and/or initiation of antibiotic treatment based on the grouping. (TIF) [file pone.0261689.s001.tif]

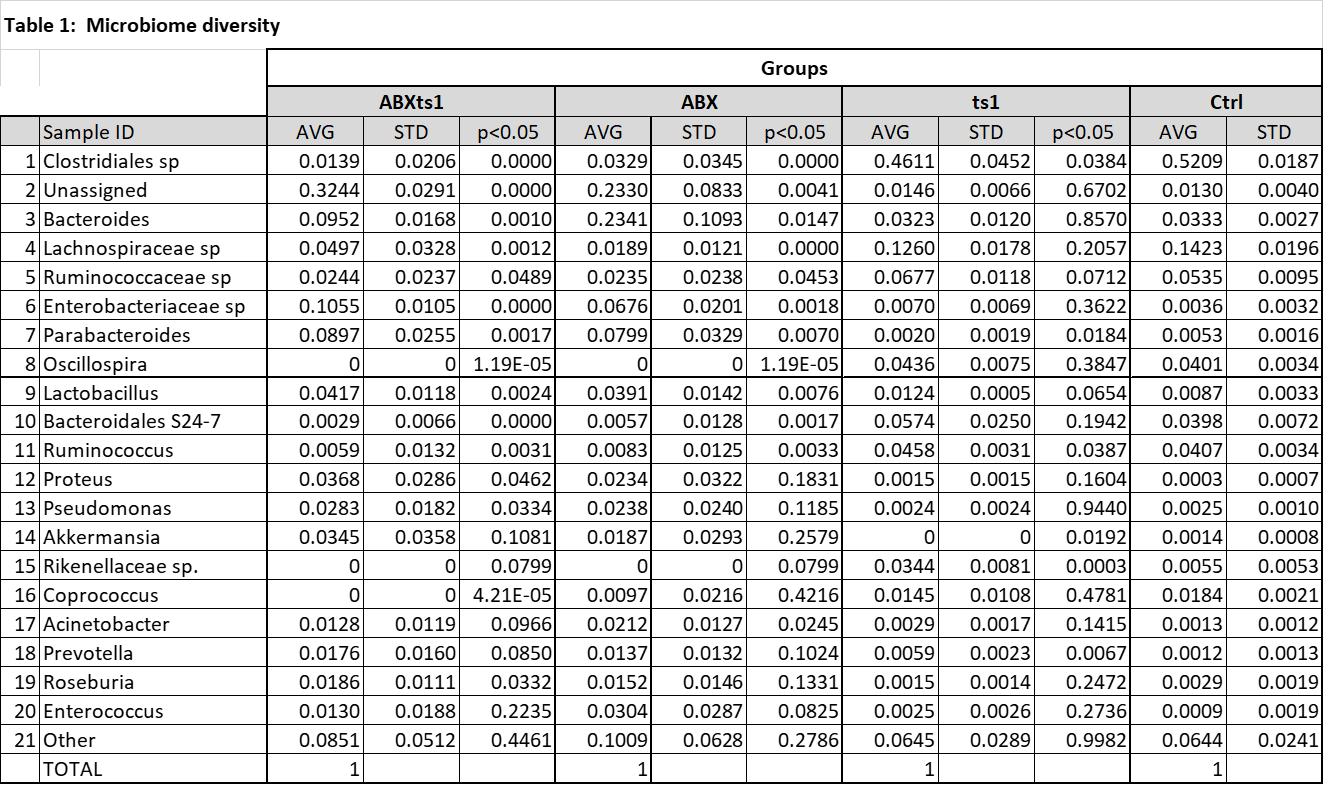

Supplement: S1 Table — Clostridiales comprise a substantial portion of the microbiome within groups not treated with antibiotics, ts1 and control. (TIF) [file pone.0261689.s002.tif]

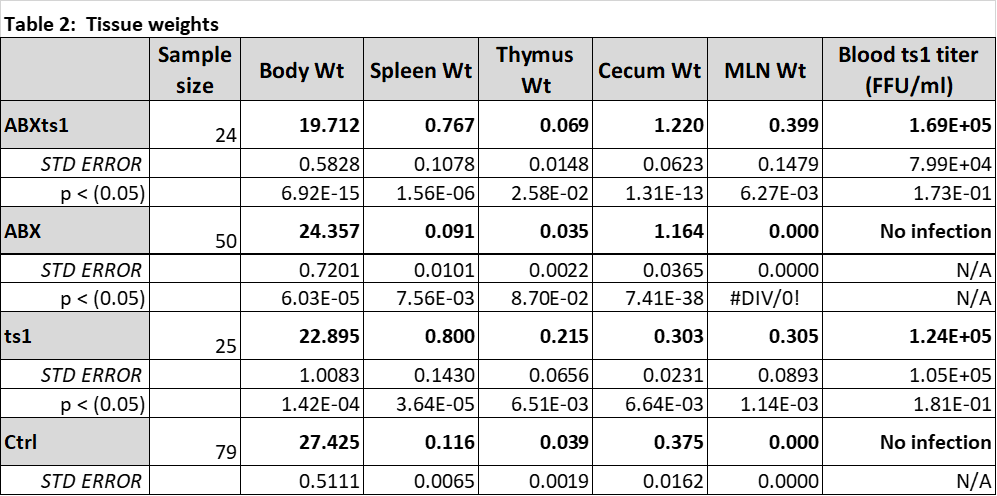

Supplement: S2 Table — Independent analogies can be inferred from the effects of the virus or the effects of the antibiotic treatment. (TIF) [file pone.0261689.s003.tif]
