# Supplementary material for: Effects of antibiotic treatment on microbiota, viral transmission and viral pathogenesis of MoMuLV ts1 infected BALB/c mice
Source: PLoS One. 2022 Jan 21;17(1):e0261689. doi: 10.1371/journal.pone.0261689 (PMC8782509; doi:10.1371/journal.pone.0261689)
Supplement: Attachment — Submitted filename: Response to Reviewers 9-3-2021.docx [file pone.0261689.s004.docx]

**Response to Reviewers:5. Review Comments to the Author

Please use the space provided to explain your answers to the questions above. You may also include additional comments for the author, including concerns about dual publication, research ethics, or publication ethics. (Please upload your review as an attachment if it exceeds 20,000 characters)**

Reviewer #2: 1.The methods section does not detail the isolation and preparation of the bacterial population for the 16S RNA-seq. Can the authors provide such details in the methods section?

2.Can the authors add statistical analysis to the figure 2. It is not clear how many replicates are used in the experiment and the statistical method for the group comparisons

3.Is there any correlation between ts1 infection or antibiotic exposure and the observed neoplasms in the study. Also can the microbial dysbiosis be correlated with the observed neoplasms in the study.


There are a number of grammatical errors which should be considered by the authors:
Below are a few examples:
Line 28: Transmission and pathophysiology of ts1 infection were (not was) not significantly…
Line 62: Sentence looks incomplete: analyses at terminal end stage for infected and age comparable uninfected mice.in
Line 62: terminal end-stage
Line 74: labeled not labelled
Line 199: Cross section should be cross-section
Line 227: alterations in normal commensal microbes decrease( not decreases) viral transmission

Reviewer #3: This study indicated the finding that transmission and pathophysiology of ts1 infection was not significantly altered by the microbial composition of the GI tract. This is an interesting study that can provide a reference for studies on Moloney Murine Leukemia Virus.

Major comments:
-Why choose temperature sensitive mutant 1 to infect animals?

-There is a lot of information describing the infection program, and it is difficult to quickly understand the author's infection program clearly. Tables or diagrams may be displayed more clearly.

-What are the changes in the cecal microbiome diversity before and after the virus infection?

-In figure 2C-F, is a significant difference analysis performed? If so, please mark the p-value.

-fig 2A, please comment on the big difference in Peripheral blood ts1 titer in the ABXts1 group. The ts1 group is the same, please comment.

-Except for the viral load in Peripheral blood and body weight, what other data can assess the pathogenicity of the virus?

Minor comments:
- define all the acronyms, like “15F” in line 92

-What is mean of “in” in line 62?

6. PLOS authors have the option to publish the peer review history of their article ([what does this mean?](https://journals.plos.org/plosone/s/editorial-and-peer-review-process#loc-peer-review-history)). If published, this will include your full peer review and any attached files.


If you choose “no”, your identity will remain anonymous but your review may still be made public.


**Do you want your identity to be public for this peer review?** For information about this choice, including consent withdrawal, please see our [Privacy Policy](https://www.plos.org/privacy-policy).

Reviewer #1: No

Reviewer #2: No

Reviewer #3: No

[NOTE: If reviewer comments were submitted as an attachment file, they will be attached to this email and accessible via the submission site. Please log into your account, locate the manuscript record, and check for the action link "View Attachments". If this link does not appear, there are no attachment files.]

While revising your submission, please upload your figure files to the Preflight Analysis and Conversion Engine (PACE) digital diagnostic tool, <https://pacev2.apexcovantage.com/>. PACE helps ensure that figures meet PLOS requirements. To use PACE, you must first register as a user. Registration is free. Then, login and navigate to the UPLOAD tab, where you will find detailed instructions on how to use the tool. If you encounter any issues or have any questions when using PACE, please email PLOS at [figures@plos.org](mailto:figures@plos.org). Please note that Supporting Information files do not need this step.

Reviewer #2:

Reviewer #2: 1. The methods section does not detail the isolation and preparation of the bacterial population for the 16S RNA-seq. Can the authors provide such details in the methods section?

Additional details added. Lines 125-143.

2.Can the authors add statistical analysis to the figure 2. It is not clear how many replicates are used in the experiment and the statistical method for the group comparisons

(P < 0.05) values added to graph. Additional details can be found on lines 182-197. Table 2 added to the Supporting Information files and caption added at the end of the manuscript.

3.Is there any correlation between ts1 infection or antibiotic exposure and the observed neoplasms in the study. Also can the microbial dysbiosis be correlated with the observed neoplasms in the study.

Antibiotic exposure does not seem to affect neoplasms initiated by ts1 infection. In addition, microbial dysbiosis does not alter organ pathologies already identified in previous studies such as megacolon, splenomegaly etc. Each entity appears to produce associated pathologies independent of the other experimental condition applied and in combined tests, produced combined effects. 184-205. Table 2 contains weight changes illustrating these associated pathologies and is added to the Supporting Information files as indicated above (Q2).

There are a number of grammatical errors which should be considered by the authors:
Below are a few examples:
Line 28: Transmission and pathophysiology of ts1 infection were (not was) not significantly…

Corrected. Line 28
Line 62: Sentence looks incomplete: analyses at terminal end stage for infected and age comparable uninfected mice.in

Sentence changed. Line 63-65
Line 62: terminal end-stage

Sentence changed. Line 65
Line 74: labeled not labelled

Corrected. Line 83
Line 199: Cross section should be cross-section

Corrected. Line 226
Line 227: alterations in normal commensal microbes decrease (not decreases) viral transmission

Corrected. Line 253

Reviewer #3:

Reviewer #3: This study indicated the finding that transmission and pathophysiology of ts1 infection was not significantly altered by the microbial composition of the GI tract. This is an interesting study that can provide a reference for studies on Moloney Murine Leukemia Virus.

Major comments:
-Why choose temperature sensitive mutant 1 to infect animals?

Previous published experiments have established this mutant as consistent in producing the symptoms of interest for comparative study.

-There is a lot of information describing the infection program, and it is difficult to quickly understand the author's infection program clearly. Tables or diagrams may be displayed more clearly.
Rewritten Lines 68-103. Figure 4 flowchart added to the Supporting Information files and caption added at the end of the manuscript to further clarify the experimental design.

-What are the changes in the cecal microbiome diversity before and after the virus infection?
Addressed in lines 146-171. Table 1 added to the Supporting Information files and caption added at the end of the manuscript. Control group (Ctrl) serves as the normal microbiome diversity and the basis by which the other groups are compared.
-In figure 2C-F, is a significant difference analysis performed? If so, please mark the p-value.
p-value marked on Figure 2 and an additional table added (Table 2) to the Supporting Information files and caption added at the end of the manuscript.
-fig 2A, please comment on the big difference in Peripheral blood ts1 titer in the ABXts1 group. The ts1 group is the same, please comment.
Peripheral viral load varies widely between infected mice, but the eventual development of neoplasms is consistent. The two groups (ABXts1 and ts1) were confirmed to be positive for the presence of the virus. 184-189, 262-268. The other two (ABX and Ctrl) were negative for the virus.
-Except for the viral load in Peripheral blood and body weight, what other data can assess the pathogenicity of the virus?
The classic presentation of pathogenicity for this virus are splenomegaly, thymus hypertrophy and mesenteric lymphadenopathy (Figure 3). Lines 214-245
Minor comments:
- define all the acronyms, like “15F” in line 92
15F cells defined and referenced (#10, #11). Lines 108-113

-What is mean of “in” in line 62?

Typographical error. Line 65

Journal Requirements

1. Please ensure that your manuscript meets PLOS ONE's style requirements, including those for file naming. The PLOS ONE style templates can be found at

https://journals.plos.org/plosone/s/file?id=wjVg/PLOSOne_formatting_sample_main_body.pdf and

<https://journals.plos.org/plosone/s/file?id=ba62/PLOSOne_formatting_sample_title_authors_affiliations.pdf>

a. This has been done.

2. As part of your revisions please update your Methods to address the following: (1) if applicable: administration of anesthetics, analgesics, antibiotics, etc. during the study; (2) if applicable: unanticipated adverse events involving animals; and (3) monitoring parameters. Thank you for your cooperation in this matter

a. This has been updated. Mice were euthanized based on guidelines approved by the American Veterinary Medical Association (AVMA) panel on euthanasia.

3. Please include your full ethics statement in the ‘Methods’ section of your manuscript file. In your statement, please include the full name of the IRB or ethics committee who approved or waived your study, as well as whether or not you obtained informed written or verbal consent. If consent was waived for your study, please include this information in your statement as well.

a. This is an animal study. IACUC approval was obtained. Line 68
